# Relative hyperglycemia is associated with complications following an acute myocardial infarction: a post-hoc analysis of HI-5 data

**DOI:** 10.1186/s12933-017-0642-3

**Published:** 2017-12-12

**Authors:** Tien F. Lee, Morton G. Burt, Leonie K. Heilbronn, Arduino A. Mangoni, Vincent W. Wong, Mark McLean, N. Wah Cheung

**Affiliations:** 1 School of Medicine, Flinders University, Adelaide, Australia; 2 Southern Adelaide Diabetes & Endocrine Services, Repatriation General Hospital, Daw Park, Adelaide, SA 5041 Australia; 3 The University of Adelaide, Adelaide, Australia; 4 Centre for Diabetes & Endocrinology Research, Westmead Hospital, Sydney, Australia

**Keywords:** Stress hyperglycemia, Myocardial infarction, Insulin, Cardiac complication

## Abstract

**Background:**

Hyperglycemia is associated with increased morbidity and mortality in patients with an acute myocardial infarction (AMI). We evaluated whether complications after AMI are associated with absolute or relative glycemia.

**Methods:**

A total of 192 patients with AMI were randomized to intensive or conventional insulin therapy. Absolute glycemia was defined as mean blood glucose level (BGL) during the first 24 h following randomization. Relative glycemia was defined by the stress hyperglycaemia ratio (SHR), calculated as mean BGL divided by average glucose concentration over the prior 3 months estimated from glycosylated haemoglobin. The primary endpoint was a “complicated AMI”, defined as an AMI complicated by death, congestive cardiac failure, arrhythmia, cardiac arrest, reinfarction, cardiogenic shock, inotrope use or emergency revascularization.

**Results:**

There was not a significant association between mean BGL and complicated AMI (odds ratio (OR) 1.05 per mmol/L glucose increment, 95% confidence intervals (CI) 0.93–1.19). In contrast, SHR was positively associated with a complicated myocardial infarction (OR 1.22 per 0.1 SHR increment, 95% CI 1.06–1.42), and individual complications of death (OR 1.55, 95% CI 1.14–2.11), congestive cardiac failure (OR 1.27, 95% CI 1.05–1.54), arrhythmia (OR 1.31, 95% CI 1.12–1.54) and cardiogenic shock (OR 1.42, 95% CI 1.03–1.97). The relationship between SHR and a complicated AMI was independent of diabetic status, intensive insulin therapy, sex and hypoglycemia.

**Conclusions:**

Relative, but not absolute, glycemia during insulin treatment is independently associated with complications after an AMI. Future studies should investigate whether basing therapeutic glycaemic targets on relative glycemia improves patient outcomes.

## Background

Hyperglycemia is associated with increased morbidity and mortality in hospitalized patients with a variety of medical conditions [1–3]. However, while the association between glucose concentration in hospital and mortality is strong in patients without known diabetes, paradoxically glucose concentration is not as strongly associated with mortality in patients with diabetes [2–6]. This suggests that background glycemia influences the relationship between glucose and mortality in patients admitted to hospital.

An elevated blood glucose in a hospitalized patient can occur because a patient has poor chronic glycemic control or if there is an acute increase in glucose, often termed stress hyperglycemia [7]. Stress hyperglycemia is the relative increase in glucose in response to an intercurrent illness. Our group has recently proposed a novel metric for relative glycemia termed the *stress hyperglycemia ratio* (SHR), whereby admission glucose concentration is corrected for background glycemia estimated from glycated hemoglobin (HbA_1c_) [8]. We reported that SHR was associated with critical illness in hospitalized patients, independent of absolute glycemia. Moreover, the association between relative glycemia and critical illness was present in patients with and without background hyperglycemia. Since this publication, several other studies have reported that relative glycemia at hospital admission predicts outcomes for patients admitted to hospital with a stroke [9], acute illness [10], acutely ill requiring intensive care unit (ICU) admission [11] and after percutaneous coronary intervention [12]. These studies demonstrate that quantifying relative hyperglycemia at admission to hospital provides important prognostic information in patients with and without diabetes.

Calculation of relative hyperglycemia could also potentially provide a basis for individualized glycemic targets in patients treated with insulin in hospital. If relative hyperglycemia during glucose-lowering treatment was associated with adverse patient outcomes, it would support this hypothesis. However, in our previous study blood glucose levels (BGLs) were not systematically recorded in all patients throughout the hospital admission and this analysis could not be undertaken [8].

The hyperglycemia: intensive insulin infusion in infarction (HI-5) study was a prospective randomized-controlled trial investigating the effect of intensive insulin therapy on mortality in patients with an acute myocardial infarction [13]. In the primary analysis, there was no significant difference in mortality in patients randomized to intensive insulin and conventional care. However, as finger prick BGLs were systematically recorded in both groups, the study cohort provides an opportunity to investigate the association between relative hyperglycemia during glucose-lowering treatment and adverse patient outcomes.

We hypothesized that, in the HI-5 study cohort, relative hyperglycemia during the first 24 h following AMI would be more strongly associated with an adverse outcomes for patients than absolute glycemia. If true, this would provide supportive evidence for a change in treatment paradigm whereby glucose-lowering treatment in patients following an AMI is targeted at relative, rather than absolute, hyperglycemia. Therefore, the aim of this study was to assess the relationship between relative glycemia and complications following a myocardial infarction and whether this relationship was affected by other potential confounding factors.

## Methods

### Study design

This is a secondary analysis of a prospective randomized-controlled trial that has previously been reported [13]. In brief, consecutive consenting patients presenting with an AMI at six hospitals in New South Wales, Australia with either known diabetes or without diabetes and BGL > 7.8 mmol/L were randomized to receive insulin/dextrose infusion therapy for at least 24 h to maintain finger prick BGL between 4 and 10 mmol/L or conventional therapy comprising their usual glucose-lowering therapy except metformin and supplemental subcutaneous short-acting insulin if finger prick BGL was ≥ 16 mmol/L. BGL were recorded at 8 pre-defined timepoints in all patients (0700, 0900,1200, 1400, 1700, 1900, 2200 and 0300 in all patients). Additional BGLs were performed on an hourly basis in patients on intensive insulin therapy. The study was approved by local ethics committees of Westmead Hospital, Nepean Hospital, Blacktown Hospital, Mt. Druitt Hospital and John Hunter Hospital. All subjects participating in the study provided written informed consent.

### Subjects

This analysis included all participants in the HI-5 study in whom HbA_1c_ was measured at admission to hospital. Patients were treated with angioplasty, thrombolysis or anti-coagulation at the discretion of the admitting doctors. All patients received beta blocker therapy unless specifically contraindicated [13]. Demographic data, laboratory data, and in-hospital mortality and complications that were recorded at the time of the original study were used in the analysis. A Charlson Comorbidity Index was calculated from the co-morbidities recorded during the original data collection [14].

### Calculation of stress hyperglycemia ratio

Finger prick point of care capillary BGLs were measured at predefined time points during the first 24 h as previously described [13], and the readings were averaged to calculate mean BGL during glucose-lowering treatment for each subject. Estimated average glucose over the prior 3 months was calculated using the equation “estimated average glucose = (1.59 × HbA_1c_) − 2.59” derived by Nathan et al. [15] Relative hyperglycemia during glucose-lowering treatment (SHR) was then calculated using the formula mean BGL divided by estimated average glucose.

### Statistical methods

Before undertaking this secondary analysis we pre-specified a composite primary endpoint of a “complicated AMI,” defined as AMI complicated by death during the hospital admission, congestive cardiac failure, arrhythmia, cardiac arrest, reinfarction, cardiogenic shock, inotrope use or the need for rescue percutaneous transluminal coronary angioplasty (PTCA) or emergency coronary arterial bypass graft (CABG). These events were defined as per the original HI-5 study [13]. Reinfarction was defined as a new AMI that occurred at least 72 h following the index AMI. A patient was considered in congestive cardiac failure if there was documented dyspnea in the notes and the chest X-ray report confirmed pulmonary edema or interstitial edema. Cardiogenic shock referred to a state where the patient was in cardiac failure with a concomitant systolic blood pressure of less than 80 mmHg. The primary variables of interest were absolute hyperglycemia, defined as mean BGL during glucose-lowering treatment and relative hyperglycemia, defined as SHR during glucose-lowering treatment.

Characteristics of patients with and without a complicated AMI are reported as mean ± standard deviation if normally distributed and median (interquartile range) if the distribution was not normal. These variables were compared using unpaired t tests, Mann–Whitney U tests or Chi squared tests as appropriate. Univariable binomial logistic regression analyses were undertaken to calculate the odds ratio of a complicated AMI for each BGL and SHR increment. Odds ratios for the individual components of the composite primary endpoint for each BGL and SHR increment were also calculated. We then included treatment group and known diabetes and interaction terms for these variables as co-variates in analyses to assess whether they moderated the relationship between SHR and a complicated AMI. Finally, univariable binomial logistic regression analyses were undertaken to examine whether other variables of interest such as sex, age, Charlson Comorbidity Index, hypoglycemia, treatment type and peak creatinine phosphokinase were associated with a complicated AMI. If a variable was not normally distributed, it was log-transformed to achieve a normal distribution before inclusion in regression analyses. Variables that were significantly associated with a complicated AMI in a univariable analysis were then included in a multiple binomial logistic regression analysis.

Statistical analysis was undertaken using SPSS version 23 for Windows (IBM, New York, USA). A two-tailed p value of < 0.05 was considered statistically significant.

## Results

### Patient characteristics

A total of 192 subjects were included in the final analysis; 48 patients out of the 240 patients included in the original study cohort were excluded as there were no recorded HbA_1c_. In this cohort, 82 patients were defined as having a complicated AMI: 6 patients died, 22 developed congestive cardiac failure, 48 had an arrhythmia, 21 had a cardiac arrest, 3 had a re-infarction, 5 developed cardiogenic shock, 10 were treated with inotropic support, 26 required a rescue PTCA and 7 an emergency CABG. A number of subjects had more than one complication. Although subjects with a complicated AMI were more likely to be female, there were no significant differences in age, Charlson Comorbidity Index, intensive insulin therapy, known diabetes, other cardiovascular risk factors, cardiovascular medications, serum cholesterol, peak creatinine phosphokinase and use of anticoagulant, thrombolytic and angioplasty treatment and ST elevation on ECG between patients with a complicated and uncomplicated AMI (Table 1).

**Table 1:** Characteristics of patients with and without a complicated myocardial infarction

	Complicated AMI	Uncomplicated AMI	p value
Number	82	110	
Age (years)	63 (10)	61 (12)	0.108
Female [N, (%)]	24 (29)	16 (15)	0.017
Charlson Comorbidity Index^a^	0.5 (0.0–1.0)	0.0 (0.0–1.0)	0.843
Intensive insulin [N, (%)]	44 (54)	58 (53)	0.898
Diabetes [N, (%)]	38 (46)	53 (48)	0.801
Diagnoses prior to admission			
Hypertension [N, (%)]	50 (61)	57 (52)	0.287
Hyperlipidemia (N, (%))	44 (54)	67 (61)	0.388
Smoker [N, (%)]	26 (32)	35 (32)	0.372
Previous AMI [N, (%)]	20 (24)	21 (19)	0.445
Medications at admission			
ACE inhibitor [N, (%)]	16 (20)	23 (21)	0.845
Aspirin [N, (%)]	19 (23)	28 (25)	0.752
Beta-blocker [N, (%)]	17 (21)	13 (12)	0.098
CCB [N, (%)]	9 (11)	13 (12)	0.880
Fibrate [N, (%)]	1 (< 1)	1 (< 1)	0.826
Nitrate [N, (%)]	11 (13)	16 (15)	0.850
Statin [N, (%)]	22 (27)	31 (28)	0.918
Initial treatment			0.583
LMWH/heparin [N, ((%)]	22 (27)	39 (35)	
Thrombolysis [N, (%)]	33 (40)	20 (18)	
Acute PTCA [N, (%)]	24 (29)	49 (45)	
Not documented	3 (4)	2 (2)	
Total cholesterol (mmol/L)	4.67 (1.31)	4.92 (1.23)	0.197
Peak CPK^a^	923 (292–3130)	1115 (517–2844)	0.305
STEMI [N, (%)]^b^	62 (76%)	84 (76%)	0.780

*ACE* angiotensin converting enzyme, *AMI* acute myocardial infarction, *N* number of patients with endpoint, *CCB* calcium channel blocker, *LMWH* low molecular weight heparin, *PTCA* percutaneous transluminal coronary angioplasty, *Peak CPK* peak creatinine phosphokinase, *STEMI* ST elevation on ECG or presence of Q-waves

Values represent mean (standard deviation) unless otherwise stated; ^a^ Median (interquartile range); ^b^ There were 6 patients missing STEMI status, 2 with uncomplicated AMI and 4 with complicated AMI

### Associations between absolute and relative glycemia during glucose-lowering treatment and a complicated acute myocardial infarction

**Fig. 1:**
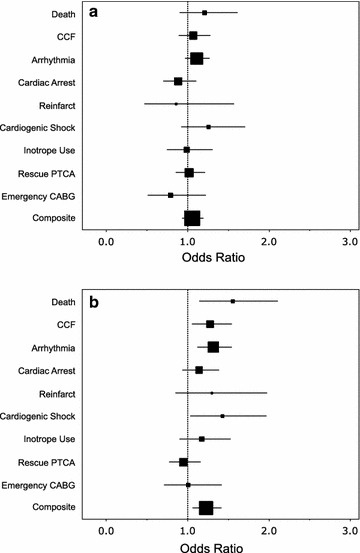
Odds ratios for the risk of complications of an acute myocardial infarction in 192 patients. **a** Complications per 1 mmol/L glucose increment and **b** 0.1 stress hyperglycemia ratio increment. The size of the squares represent the observed frequency of each complication post myocardial infarction while the whiskers represent the 95% confidence intervals. CCF = congestive cardiac failure; PTCA = percutaneous transluminal coronary angioplasty; CABG = coronary arterial bypass graft

There was not a significant association between mean BGL and the incidence of complicated AMI (odds ratio (OR) 1.05 per mmol/L glucose increment, 95% confidence intervals (CI) 0.93–1.19, p = 0.437) (Fig. 1a). Furthermore, no individual component of the composite “complicated AMI” endpoint was significantly associated with mean BGL (Fig. 1a). In contrast, SHR was associated with a complicated AMI (OR 1.22 per 0.1 SHR increment, 95% CI 1.06–1.42, p = 0.006) (Fig. 1b). Moreover, SHR was positively associated with death (OR 1.55, 95% CI 1.14–2.11, p = 0.005), congestive cardiac failure (OR 1.27, 95% CI 1.05–1.54, p = 0.014), arrhythmia (OR 1.31, 95% CI 1.12–1.54, p = 0.001) and cardiogenic shock (OR 1.42, 95% CI 1.03–1.97, p = 0.033). SHR was not significantly associated with cardiac arrest, reinfarction, inotrope use, rescue angioplasty or emergency CABG (Fig. 1b).

### Effect of diabetes and intensive insulin therapy on association between relative glycemia and a complicated acute myocardial infarction

**Fig. 2:**
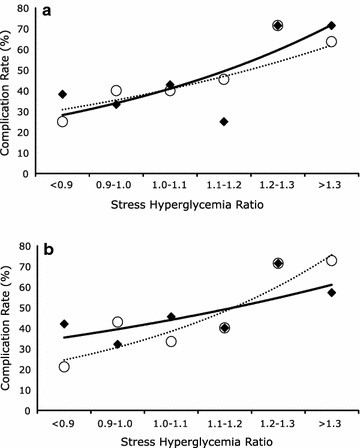
Rates of a complicated acute myocardial infarction per stress hyperglycemia ratio range in 192 patients. **a** Patients with (black diamond, solid line) and without (white circle, dotted line) Diabetes Mellitus; and **b** patients receiving intensive (black diamond, solid line) or conventional (white circle, dotted line) glucose-lowering therapy

Figure 2a shows that the relationship between SHR and a complicated AMI was similar in patients with and without diabetes. The association between SHR and a complicated AMI was independent of diabetes status (OR 1.38 per 0.1 SHR increment, 95% CI 1.08–1.75 p = 0.009), which was not independently associated with a complicated AMI (p = 0.204). Furthermore there was not a significant interaction between SHR and diabetes status (p = 0.216), demonstrating that diabetes status did not significantly modulate the relationship between SHR and a complicated AMI. Figure 2b shows that the relationship between SHR during insulin treatment and a complicated AMI was not different in patients randomized to intensive insulin and conventional therapy. The association between SHR and a complicated AMI was independent of intensive insulin therapy (odds ratio = 1.37 per 0.1 SHR increment, 95% CI 1.09–1.72, p = 0.006), which was not associated with a complicated AMI (p = 0.173). Moreover there was not a significant interaction between SHR and intensive insulin treatment (p = 0.206), demonstrating that treatment group not significantly modulate the relationship between SHR and a complicated AMI.

### Effect of other variables on a complicated acute myocardial infarction

In univariate analyses, female sex (p = 0.015) and hypoglycemia (p = 0.015) were significantly associated with a greater risk of a complicated AMI (Table 2). Age, Charlson Comorbidity Index, treatment of the AMI, peak creatinine phosphokinase and ST elevation on ECG were not significantly associated with a complicated AMI. In a multiple regression analysis the relationship between SHR and a complicated AMI was independent of sex and hypoglycemia (Table 2). Female sex and hypoglycemia were also independently associated with a complicated myocardial infarction.

**Table 2:** Univariate and multivariate analyses of associations between selected variables and a complicated acute myocardial infarction

Variable	Univariate		Multivariate	
	β	p value	β	p value
Sex	− 0.888	0.015	− 0.917	0.017
Age	0.021	0.108	NA	
SHR	0.202	0.006	0.245	0.002
Charlson Score^a^	1.105	0.740	NA	
Hypoglycemia	1.109	0.049	1.218	0.038
Treatment^b^	− 0.094	0.590	NA	
Peak CPK^a^	− 0.382	0.169	NA	
STEMI^c^	0.102	0.779		

*NA* not assessed, *SHR* stress hyperglycemia ratio, *Peak CPK* peak creatinine phosphokinase, *STEMI* ST elevation on ECG or presence of Q-waves

^a^Log transformed for statistical analysis

^b^Treatment groups were heparin or low-molecular weight heparin, thrombolysis or percutaneous transluminal coronary angioplasty

^c^There were 6 missing STEMI status which were excluded in this analysis

## Discussion

Our secondary analysis of the HI-5 study observed no significant association between absolute glycemia during glucose-lowering treatment, defined as the mean finger prick BGL during the first 24 h post-AMI, and a complicated AMI. In contrast, when mean BGL was corrected for background glycemia to estimate relative glycemia, it was positively associated with a complicated AMI. Moreover, the association with relative glycemia was independent of diabetes status, treatment group and a number of other variables that potentially affect the prognosis after an AMI. As relative glycemia during glucose-lowering treatment is more strongly associated with patient outcomes than absolute glycemia, we hypothesize that defining individualized therapeutic glucose targets based on relative, and not absolute, glycemia could potentially reduce morbidity and mortality after an AMI.

### Absolute hyperglycemia and mortality

A number of studies have reported that glucose concentration on admission to hospital is positively associated with mortality in patients with an AMI [1, 3, 16–19]. Moreover, the association between mortality and mean glucose during hospitalization is even stronger than with admission glucose [16, 20–22]. In contrast, in the HI-5 patient cohort mean glucose concentration during glucose-lowering treatment was not significantly associated with a complicated AMI or any individual component of this composite endpoint. There are a number of potential explanations for this negative result. Firstly, approximately half the patients in the HI-5 study had diabetes and the association between glucose concentration and mortality is attenuated in this group [3, 16, 23]. Secondly, the primary endpoint of this analysis is a composite and there may be individual components of the endpoint that are not related to glucose concentration. Most importantly, the sample size in this analysis is relatively small compared to previous studies.

### Relative hyperglycemia and mortality

Measurement of HbA_1c_ in addition to glucose has revolutionized the management of chronic hyperglycemia, where HbA_1c_ is both a diagnostic test for diabetes and the primary measurement used to guide the need for and efficacy of glucose-lowering therapy [24]. In addition to a clear relationship with microvascular disease, a lower HbA1c during long-term follow-up has been associated with reduced macrovascular disease [25, 26]. It has also been hypothesized that HbA_1c_ could be used to quantify stress or relative hyperglycemia and assist management of acute hyperglycemia [27, 28]. However, other studies have utilized varying definitions of stress hyperglycemia based on absolute glucose concentrations, sometimes taking a patients’ diabetic status into account [20, 29, 30]. While it has been proposed that therapeutic targets should differ in patients with and without diabetes, the optimum method to quantify relative glycemia remains to be determined [28, 31]. SHR is a novel metric to quantify relative glycemia in a single numerical value that is cheap to measure and simple to calculate. It directly relates a patient’s current glucose control during an acute presentation to their background glycemia, providing an individualized quantification of stress or relative hyperglycemia.

In contrast to absolute glycemia, relative glycemia during glucose-lowering treatment was positively and significantly associated with mortality, heart failure, arrhythmia, cardiogenic shock and a composite endpoint of a complicated AMI in the HI-5 study. These associations between relative glycemia and a number of adverse patient outcomes are striking, given the fairly small sample size in this analysis. Our study extends previous observations by demonstrating that relative hyperglycemia during glucose-lowering treatment is also associated with adverse outcomes in patients with and without diabetes. This suggests that a management strategy that selectively targets glucose-lowering therapy for patients with an elevated SHR irrespective of prior diabetes status may improve outcomes after an AMI.

Others have reported that relative hyperglycemia at admission to hospital has been associated with poor outcomes in patients with acute illness [10], stroke [9], acute myocardial infarction [30, 32] and in cardiogenic shock [33]. In addition to SHR two other measures of relative glycemia have been proposed. The “glycemic gap” is the difference between the admission glucose and the estimated average glucose i.e. it is the absolute rather than the relative difference in glucose concentration [11, 32, 34]. Another proposal was for a “glucose concentration-to-HbA1c ratio”, in which the admission glucose is divided by the HbA1c [9]. This is theoretically similar to SHR. Whether one measure has advantages over the others has yet to be fully determined, although they are likely perform similarly [9].

### Effects of diabetes status and insulin use

During long-term follow-up of patients with AMI diabetes is associated with increased mortality [35, 36]. However, during a hospital admission the association between absolute glycemia and mortality is stronger in patients without, as opposed to with, diabetes [3, 6, 18]. We previously reported that the relationship between relative glycemia and critical illness was similar in patients with and without background hyperglycemia [8]. In this analysis, relative glycemia was also associated with a complicated AMI independent of diabetes, which was not independently associated with a complicated AMI (Fig. 2a). This suggests that relative hyperglycemia is clinically important regardless of diabetes status.

A potential confounder in this analysis is that half the cohort was randomized to intensive insulin therapy, which will lower absolute, and consequently relative, glycemia. However, the 0.7 mmol/L difference in absolute glucose between the two groups was not statistically significant [13]. Furthermore, the positive association between relative glycemia and a complicated AMI was independent of treatment group. Treatment group itself was not independently associated with a complicated AMI and there was not a significant interaction between treatment group and SHR in regression analysis. This suggests that insulin treatment did not confound the relationship between relative glycemia and a complicated AMI.

### Other variables affecting outcomes

The other variables that were independently associated with a complicated AMI were female sex and hypoglycemia. The association between female sex and a poorer outcome after an AMI is consistent with previous studies [37, 38]. An association between hypoglycemia after an AMI and mortality is well described [16, 19, 39]. However, previous studies have reported that the association is predominantly in patients with spontaneous hypoglycemia while not on insulin therapy and at admission to hospital [19, 39]. In our study, hypoglycemia during glucose-lowering treatment was independently associated with a complicated AMI. This suggests that if a therapeutic approach targeting relative glycemia were to be trialled, it will be important to avoid hypoglycemia.

Some variables that are usually associated with poor outcomes after AMI were not statistically significant in our analyses. ST elevation was not a predictor of complicated AMI. Our analysis cannot determine the reason for this, but we postulate contributory factors include that only patients with STEMI were revascularized, the primary endpoint was composite and that the sample size was relatively small. The association between age and complicated AMI was positive but did not reach statistical significance (p = 0.108). Peak CK also did not show an association with poorer composite outcomes. Only peak CK was measured and recorded, as CKMB and troponin measurements were not routine at the time of the HI-5 study. Other variables can affect peak CK, such as procedures like PTCA.

### Mechanisms linking stress hyperglycemia and patient outcomes

An elevation in plasma glucose concentration is potentially an epiphenomenon and not directly contributing to cardiovascular events. For example, patients with an AMI have elevations of serum cortisol that could mediate both an increase in blood glucose and contribute to increased cardiovascular risk [40–42]. However, a number of mechanisms have been proposed by which stress hyperglycemia could directly increase morbidity and mortality after an AMI. These include endothelial apoptosis, endothelial dysfunction and oxidative stress [43–46]. Interventional studies demonstrating that lowering plasma glucose reduces cardiovascular events are needed to confirm that stress hyperglycemia per se increases cardiovascular risk.

Studies that have lowered glucose concentration to a target absolute glucose range using insulin therapy have produced conflicting results [13, 47, 48]. Consequently, the importance of this therapeutic approach has not been clearly defined, supported only by a low-level of evidence in clinical practice guidelines [20, 49, 50]. As relative glycemia during glucose-lowering treatment is more strongly associated with adverse outcomes, we propose that SHR could be used to derive individualized glycemic targets for patients with an AMI. If this improved patient outcomes it would represent a paradigm shift in the management of hyperglycemia in hospitalized patients. Treatment of relative hyperglycemia should be the subject of future interventional studies.

### Strengths and limitation

The strengths of this study include the use of a novel metric that quantifies stress hyperglycemia as a continuous variable, a patient population that reflects usual clinical practice, patient data that was systematically and prospectively recorded, and defining a pre-specified primary endpoint before undertaking the analysis. However, we acknowledge that the study has limitations. This is a retrospective, post hoc analysis, and is thus hypothesis generating. The study design cannot show causality, does not assess mechanisms by which relative hyperglycemia could confer a poorer prognosis and does not distinguish whether spontaneous normalization of glucose or insulin treatment underlies a relationship between relative hypoglycemia and a complicated AMI. In particular, it cannot distinguish whether relative hyperglycemia is simply a marker of the severity or duration of an AMI, or directly contributes to poorer outcomes. Moreover, we did not assess the effect of other variables in this analysis that potentially affect cardiovascular outcomes, such as glycemic variability and postprandial hyperglycemia [51, 52]. Another potential limitation is that glucose was assessed using point of care BGLs, which may differ from gold standard laboratory measurements. However, this approach reflects usual clinical practice. Management of AMI has also changed since the HI-5 study, notably the greater use of PTCA. This could potentially affect results, although treatment was not a predictor of outcome in this analysis. The mortality rate was fairly low in this study and the results may not be able to be extrapolated to patient groups with a higher mortality rate. Finally, the sample size was relatively small, which may have resulted in a type 2 error, especially in subgroup analysis of treatment groups as the numbers of patients in the insulin-treated group with a high SHR were low. Nevertheless, the study was of sufficient size to show a significant independent association between relative glycemia and several cardiovascular endpoints.

## Conclusion

Absolute glucose concentration during glucose-lowering treatment following an AMI was not significantly associated with a complicated AMI. In contrast, SHR was positively associated with mortality, heart failure, arrhythmia, cardiogenic shock and a composite endpoint of a complicated AMI. We conclude that relative glycemia during glucose-lowering treatment is more strongly associated with adverse outcomes than absolute glycemia in patients after an AMI. This research adds to other published research by demonstrating the prognostic utility of quantifying relative hyperglycemia during glucose-lowering treatment after an AMI, and should provide a basis for prospective studies using relative, rather than absolute, glycemic thresholds for intervention and therapeutic glycemic targets.
